# Comprehensive analysis of CPSF4-related alternative splice genes in hepatocellular carcinoma

**DOI:** 10.1007/s00432-023-05178-z

**Published:** 2023-08-05

**Authors:** Anwaier Yuemaierabola, Jun Guo, Lili Sun, Buerlan Yeerkenbieke, Fuzhong Liu, Dilinaer Ye, Xiaoyi Zhai, Wenjia Guo, Yan Cao

**Affiliations:** 1 Department of Cancer Research Institute, Affiliated Cancer Hospital of Xinjiang Medical University, Urumqi, 830011 China; 2 Key Laboratory of Oncology of Xinjiang Uyghur Autonomous Region, Urumqi, 830011 China; 3 Cancer Hospital of Xinjiang Uygur Autonomous Region (Affiliated Cancer Hospital of Xinjiang Medical University), Urumqi, 830011 China; 4 Nursing School of Xinjiang Medical University, Urumqi, 830011 China

**Keywords:** Hepatocellular carcinoma (HCC), Alternative splice (AS), CPSF4, Prognosis, Immune infiltration

## Abstract

**Background:**

An important stage in controlling gene expression is RNA alternative splicing (AS), and aberrant AS can trigger the development and spread of malignancies, including hepatocellular carcinoma (HCC). A crucial component of AS is cleavage and polyadenylation-specific factor 4 (CPSF4), a component of the CPSF complex, but it is unclear how CPSF4-related AS molecules describe immune cell infiltration in the total tumor microenvironment (TME).

**Methods:**

Using RNA-sequencing data and clinical data from TCGA-LIHC from the Cancer Genome Atlas (TCGA) database, the AS genes with differential expression were found. The univariate Cox analysis, KM analysis, and Spearman analysis were used to identify the AS genes related to prognosis. Screening of key AS genes that are highly correlated with CPSF4. Key genes were screened using Cox regression analysis and stepwise regression analysis, and prognosis prediction models and the topography of TME cell infiltration were thoroughly analyzed.

**Results:**

A model consisting of seven AS genes (STMN1, CLSPN, MDK, RNFT2, PRR11, RNF157, GHR) was constructed that was aimed to predict prognostic condition. The outcomes of the HCC samples in the high-risk group were considerably worse than those in the lower risk group (*p* < 0.0001), and different risk patient groups were formed. According to the calibration curves and the area under the ROC curve (AUC) values for survival at 1, 2, and 3 years, the clinical nomogram performs well in predicting survival in HCC patients. These values were 0.76, 0.70, and 0.69, respectively. Moreover, prognostic signature was markedly related to immune infiltration and immune checkpoint genes expression.

**Conclusion:**

By shedding light on the function of CPSF4 and the seven AS genes in the formation and progression of HCC, this research analysis contributes to the development of more useful prognostic, diagnostic, and possibly therapeutic biomarkers.

**Supplementary Information:**

The online version contains supplementary material available at 10.1007/s00432-023-05178-z.

## Introduction

The second greatest cause of cancer-related deaths globally is hepatocellular carcinoma (Siegel et al. 2020). Hepatocellular carcinoma is now treated with surgery, chemotherapy, radiation, and liver transplantation (Chaabna et al. 2021). Nonetheless, the general prognosis of liver cancer patients is poor due to the invasion, migration, medication resistance, and uncertain diagnosis of cancer cells (Jiang et al. 2021). The advent of immunotherapy has brought prospect to patients with advanced HCC, and the tumor microenvironment (TME) in HCC has multiple capabilities that strongly influence tumor initiation and progression (Eggert and Greten 2017). A significant group of regulatory pathways known as immune checkpoints are essential for escalating inflammatory reactions and sustaining self-tolerance (Waldman et al. 2020). Advanced hepatocellular carcinoma patients have been demonstrated to benefit from immune checkpoint inhibitors (ICIs) (Sheng et al. 2020). Notwithstanding, the vast majority of patients have negligible or no clinical benefit from immune checkpoint blockade, far from meeting clinical needs (Newman et al. 2015; Angelova et al. 2015). Hence, thorough study of the pathophysiology of HCC and the identification of new therapeutic targets and prognostic biomarkers are of tremendous clinical significance for enhancing the efficiency of immunotherapy medications and enhancing the prognosis and quality of life of patients (Wang et al. 2021a).

Alternative splicing, a post-transcriptional process, produces alternative mRNA transcripts critical for normal development and contributes to the proteomic complexity of mammalian genomes (Chen et al. 2019). Growing evidence suggests that AS has become a major source of protein diversity in more than 90% of the human genome, a member of the important molecular markers of human cancer, and a promising target for novel cancer treatments (Baralle and Giudice 2017). Studies have reported that alternative splicing not only has a conspicuous correlation with tumor occurrence and development, invasion and metastasis, and treatment resistance, but also plays a noteworthy role in the formation of immune microenvironment (Qi et al. 2020). CPSF4 is a component of the CPSF complex and is essential for the maturation of the 3′-end and polyadenylation of mRNA. Recent genome-wide histone–RNA interaction studies have shown that regulation of pre-mRNA alternative splicing and alternative polyadenylation are two processes that closely are related, with 3′-end forming factors playing an vital role in alternative splicing (Misra and Green 2016), and it has also been reported that CPSF4 is implicated in the alternative splicing of Tp53 mRNA (Dubois et al. 2019). However, it is yet unknown how CPSF4 affects the onset and progression of liver cancer as well as the molecular system that controls it. More research is still required to determine whether CPSF4 can serve as a diagnostic marker or therapeutic target for liver cancer.

In this work, we investigated the biological role of CPSF4, its expression in HCC, and the prognosis of the disease. Seven key AS molecules related to CPSF4 were pointed out, and these molecules were not only related to the prognosis of hepatocellular carcinoma, but also related to expression of immune checkpoint genes and immune cell invasion. As a result of the research analysis, additional useful prognostic, diagnostic, and prospective therapeutic targets will be suggested. They include the involvement of CPSF4 and related AS molecules in the onset and progression of liver cancer.

## Materials and methods

### Data

For 371 HCC patient samples and 50 normal samples, the RNA-sequencing (RNA-seq) data and related clinical information were retrieved from the TCGA website (https://portal.gdc.cancer.gov/repository). Fifty-six HCC patient samples with a time to live greater than 6 years and missing values were excluded. To normalize the gene expression profiles, we applied the scale approach offered by the “DESeq2” R package (Love et al. 2014). Another 240 tumor samples’ RNA-seq data and clinical details were received through the ICGC portal (https://dcc.icgc.org/projects/LIRI-JP).

### Functional annotation of CPSF4 gene

In the TIMER2 database, 33 different cancer types' CPSF4 gene expression was examined (http://timer.cistrome.org/). Gene Ontology (GO) and Kyoto Encyclopedia of Genes and Genomes (KEGG) analyses were performed based on the CPSF4 expression relevant genes (|*r*| > 0.3, *p* value < 0.05), utilizing the “clusterProfiler” R program between the tumor and normal groups. The BH approach was used to change the *p* values. The R packages “survival” and “ggplot2” were used to perform the HCC survival analysis. IHC pictures of CPSF4 protein expression in healthy tissues and HCC tissues were acquired from the Human Protein Atlas (HPA) (http://www.proteinatlas.org/) to compare differences in CPSF4 expression at the protein level.

### Finding differentially expressed AS genes

Identification of differentially expressed genes (DEGs): The DESeq2 package was used to normalize the data for each gene expression profile into cpm values. Significant DEGs were selected using cutoff criteria *p* value < 0.05 and |logFC|> = 2. AS data were obtained for the identified DEGs in the TCGA Splice-seq database with sample percentages of PSI values: 100, minimum PSI range (increments between samples): 0, minimum PSI standard deviation: 0.

### Construction of AS gene signature

First, univariate Cox regression analysis for OS was used to eliminate the predictive AS genes. The predictive AS genes based on lambda.min were then combined in a multi-gene signature using LASSO regression. Via tenfold cross-validations, the ideal value of lambda was determined. Using the R packages “survival” and “glmnet”, Cox and LASSO regressions with one variable were carried out (Ramsay et al. 2018). According to each patient's signature, the risk score was determined using the formula below:

$$\mathrm{Riskscore}=\sum \mathrm{i}=1\mathrm{n\beta i}\times \mathrm{Expi}$$

where is the gene's LASSO coefficient and Exp is a representation of the gene's expression. Based on the optimal cutoff value determined by the “surv cutpoint” function of the “survminer” R package, all samples were split into groups with high and low risk. This function determines the optimal cutpoint for continuous variables using the maxstat (maximally selected rank statistics) statistic.

### Evaluation and verification of the AS gene signature

Using the R package “survivalROC”, a receiver operating characteristic curve was performed and shown to validate the discrimination of the signature. A Kaplan–Meier curve was produced using the R package “survival” to evaluate the signature's predictive ability in conjunction with a log-rank test for OS. Whether risk score was an independent predictive factor for OS in addition to the ICGC verification queue, univariate and multivariate Cox regression was used. The survival rates of the various risk groups were compared using the log-rank test and the K–M survival curve. The 1-year-to-2-year-to-3-year ROC curve was used to determine the prognostic signature's sensitivity and specificity (Heagerty et al. 2000).

### Clinical analysis of AS gene signature

The survival study verified the difference in OS between the AS gene groups with elevated and decreased expression. An analysis of the Kaplan–Meier curve and the two-side log-rank test were used in each of the aforementioned survival studies. Immunohistochemical pictures of HCC from the HPA database were examined to continue evaluating differences in AS gene protein expression levels. We performed a correlation analysis of the tumor stage with risk score and the AS gene.

### Tumor immunity analyses

The RNA-sequencing data were used to derive the expression levels of nine immune checkpoint genes thought to be potential targets for cancer immunotherapy. The expression levels of the 15 immune checkpoint genes were then compared between the two risk groups and AS genes using Wilcoxon analysis.

### Examination of AS events

To analyze AS events in AS genes, the univariate Cox analysis was used. The correlation analysis was conducted to show the connection between the CPSF4 gene expression and the PSI values of survival-associated AS events.

### Statistical analysis

Using the necessary packages, R 4.2.1 (http://www.R-project.org) carried out all of the statistical analyses. The specificity, as well as sensitivity of the developed signature, was evaluated using ROC curves. To investigate the key prognostic factors, we employed univariate and multivariate Cox regression. The Fisher exact test or 2 test was developed to analyze the association between several variables for categorical data. Measurement data between groups were compared using the Student's *t* test. Except as otherwise noted, statistical significance was defined as *p* < 0.05.

## Results

### Gene expression analysis data

**Fig. 1:**
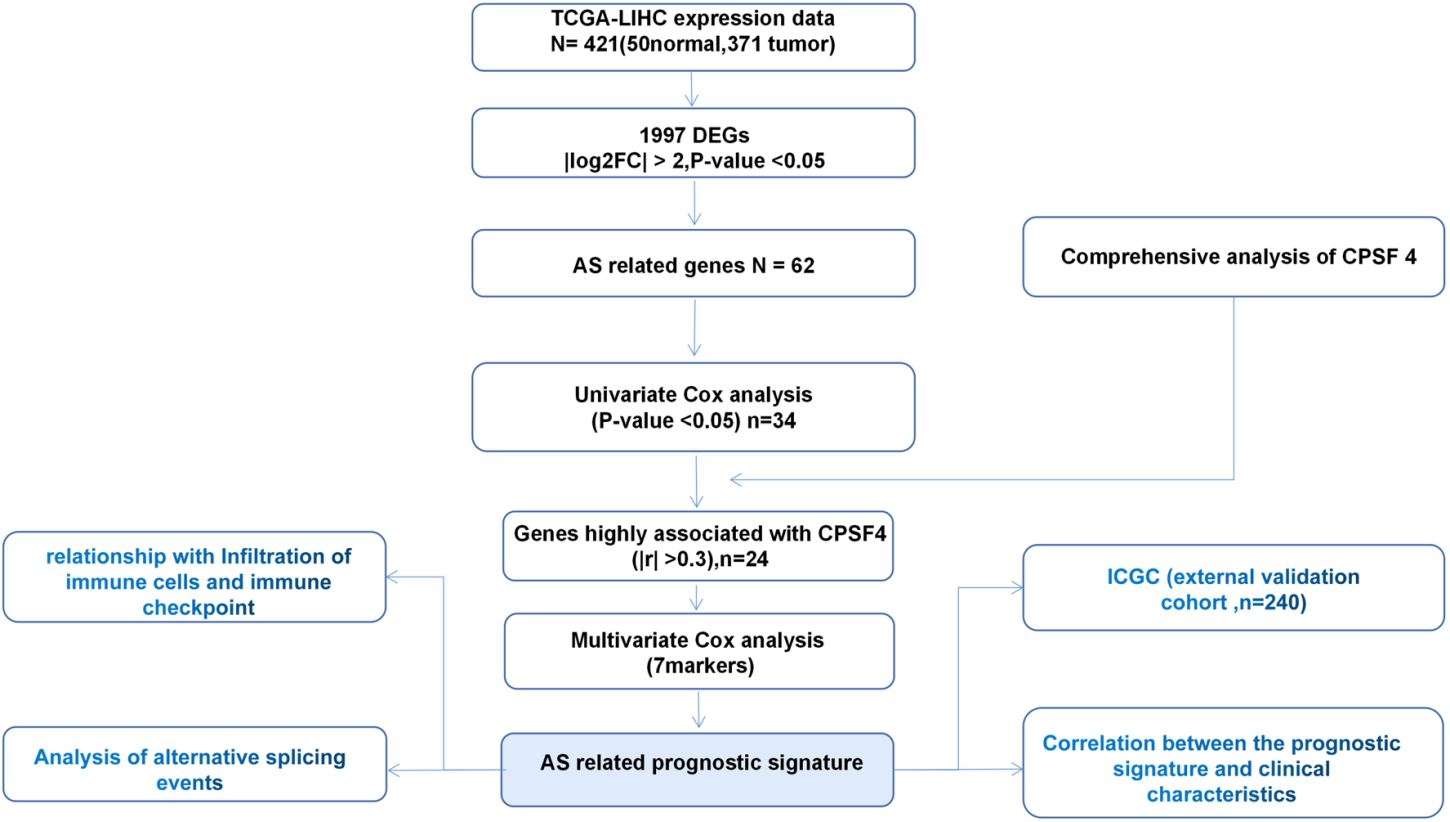
The complete data analysis flow chart

**Fig. 2:**
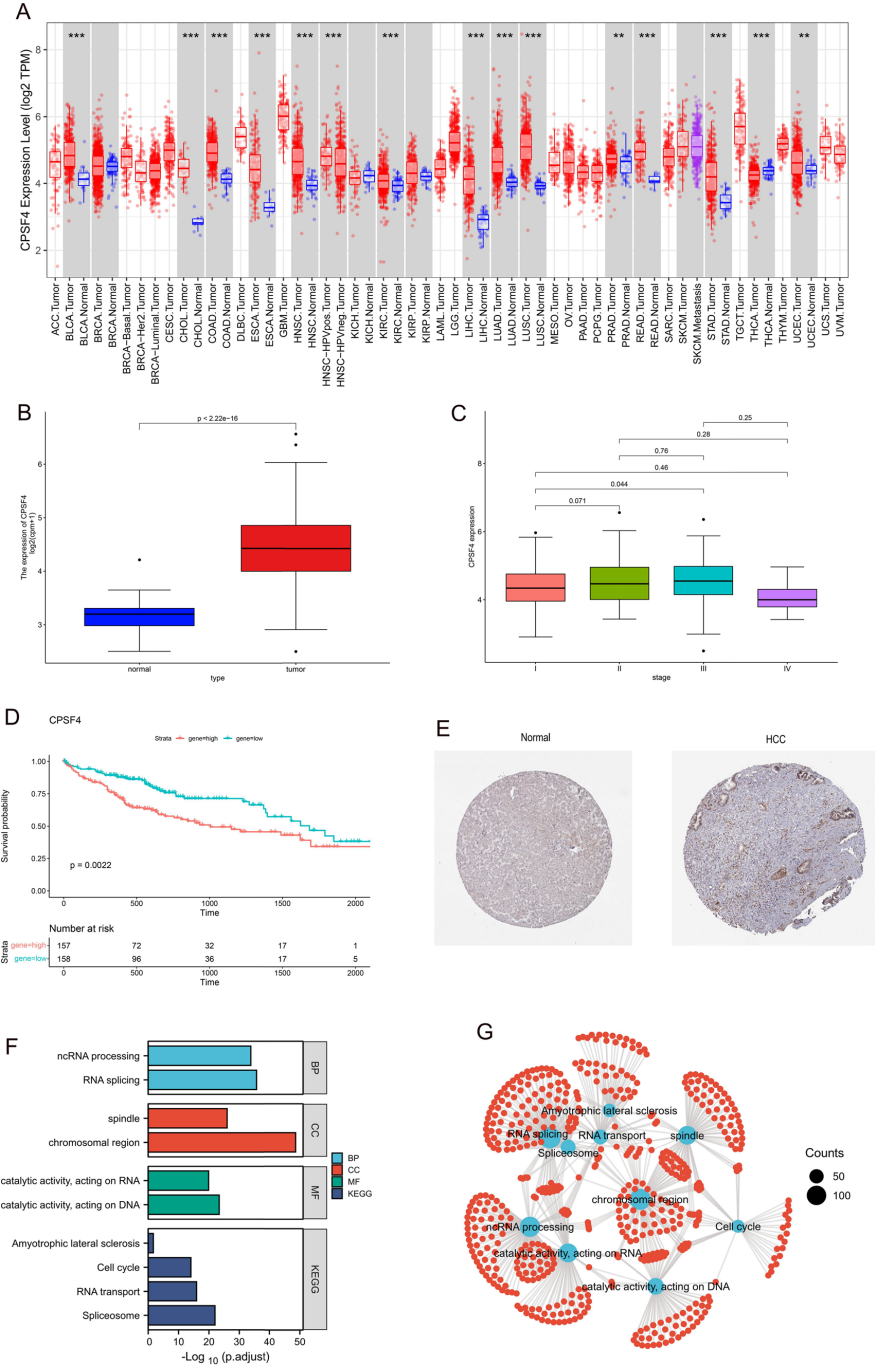
Comprehensive analysis for CPSF4 gene. **A** TIMER2's visualization of the expression of CPSF4 in TCGA tumors compared to nearby tissues (if available). *p < 0.05; **p < 0.01; ***p < 0.001. **B** Box plot depicting the comparison of CPSF4 expression levels between HCC and the equivalent normal tissues (TCGA database). **C** A clear discrepancy in the expression of CPSF4 in HCC tumors at various clinical stages. **D** Kaplan–Meier survival curves for patients with HCC malignancies stratified by CPSF4 expression levels. **E** The protein expression profiles of CPSF4 in the Human Protein Atlas database. **F**–**G** GO functional annotation and KEGG pathway analysis of CPSF4 gene

Figure 1 depicts the study's analysis procedure. Based on the TCGA database, we use the TIMER2 approach to examine the levels of CPSF4 expression in tumor tissue and nearby normal tissue in various malignancies, the results are shown in Fig. 2A. Compared to normal tissues, the expression of CPSF4 was noticeably higher in cancerous tissues (Fig. 2B); thus, we employed TCGA-LIHC RNA-sequencing data for differential expression analysis (*n* = 50 nearby normal tissues, *n* = 315 in cancer tissues). Analysis of the TCGA database's CPSF4 clinical data revealed that CPSF4 expression varied according to the clinical stage of HCC (Fig. 2C). For 315 HCC patient samples, a Kaplan–Meier OS analysis was conducted. The findings demonstrated that a worse prognosis was linked to higher expression of CPSF4 (*p* = 0.0022) (Fig. 2D). Immunohistochemical staining manifested that in HCC, CPSF4 staining was deeper in the cancerous tissue than in the corresponding paracancerous tissue. (Fig. 2E), KEGG and GO analysis showed that CPSF4 was related to cell cycle, RNA splicing, and other pathways (Fig. 2F).

### Recognition of AS genes with differential expression

**Fig. 3:**
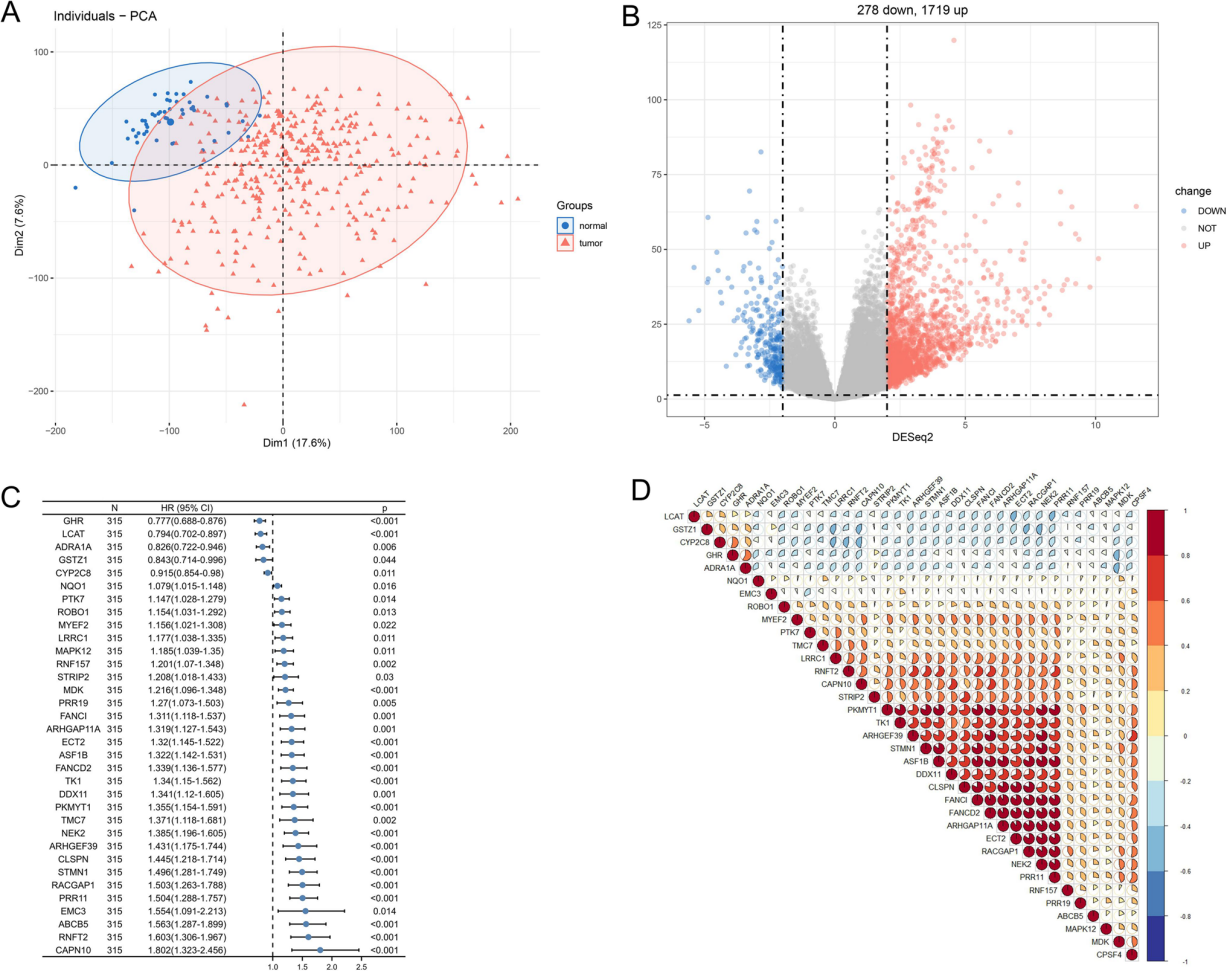
Discovery of AS genes with differential expression. TCGA cohort's **A** PCA plot. **B** A volcano plot displaying the genes in LIHC that were differently expressed. Red dots indicate 1719 upregulated genes, while 278 downregulated genes are represented by green dots. **C** A forest plot that displays the p value and confidence interval for the hazard ratios for the univariate Cox regression findings. **D** The relationship between the CPSF4 gene and the 24 AS genes relevant to survival. Blue, unfavorable positive correlation; red indicates correlation

Using the DESeq2 tool, we normalized the data for each gene expression profile. Using the threshold criteria of *p*  < 0.05 and |logFC| > = 2, significant DEGs were chosen, yielding 1719 upregulated genes and 278 downregulated genes (Fig. 3A). To reduce dimensionality and the significantly different chemical expression patterns between normal samples and HCC tumors, we applied principal component analysis (PCA) (Fig. 3B). We further obtained the AS data under the condition that DEGs were identified in the TCGA Splice-seq database, 62 AS genes were identified with 165 AS events (Table S1). Thirty-four AS genes associated with prognosis were tested at *p *< 0.05 by univariate Cox analysis (Fig. 3C). Twenty-four AS genes with a Spearman correlation coefficient |*r*|> 0.3 were chosen for further investigation and displayed the link between thirty-four AS genes and CPSF4 (Fig. 3D).

### Construction of AS-gene-related prognostic signature for HCC

**Fig. 4:**
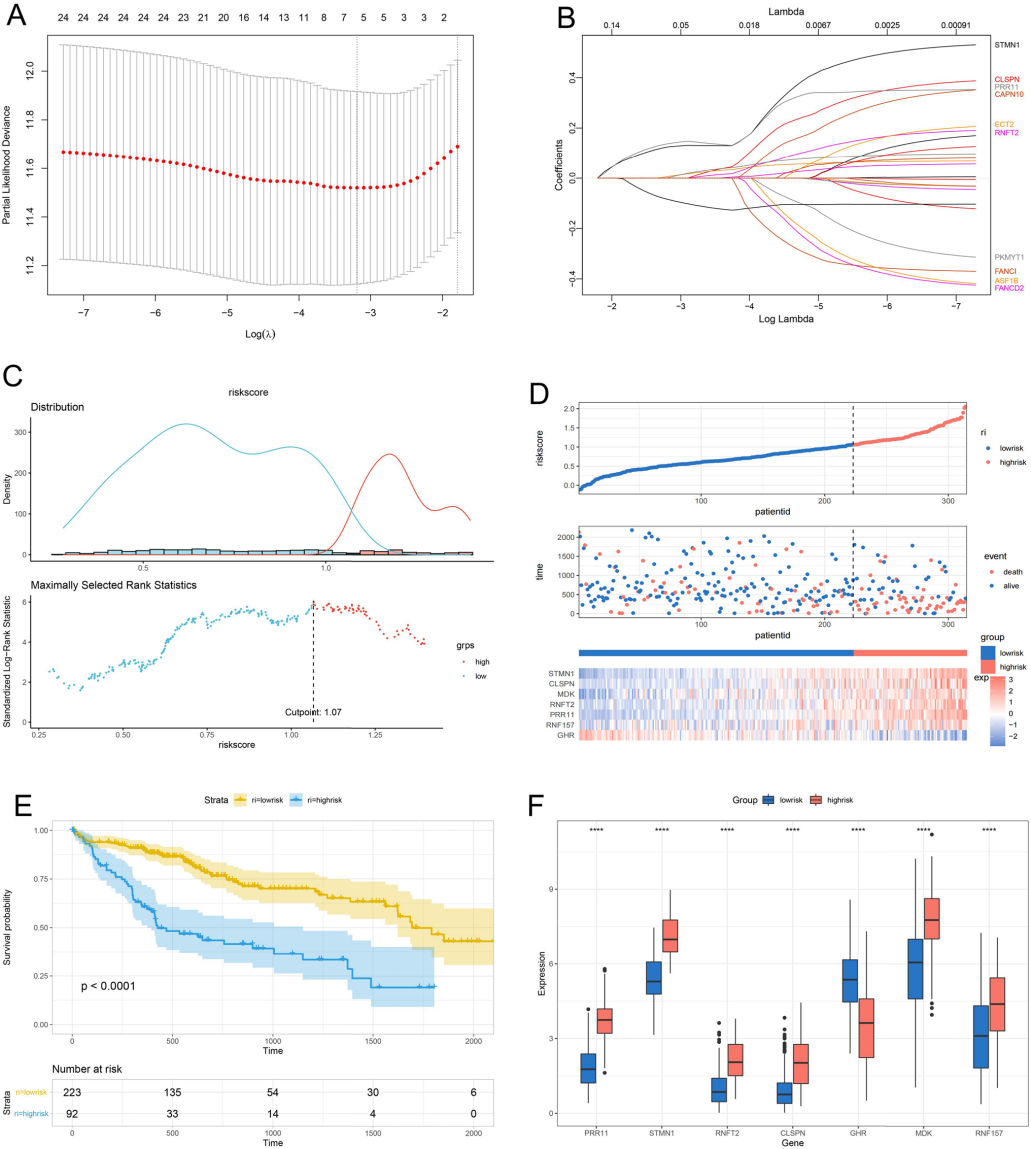
Construction of riskScore signature. **A**, **B** Lasso regression removes collinearity to identify seven AS genes. **C** The optimal cutoff point to dichotomize riskScore into low and high groups. **D** The risk score, survival status of HCC patients, heatmap of the seven AS genes expression are shown. **E** Survival curves for the low-risk and high-risk groups. **F** The seven AS genes expressed in the low- and high-risk groups (*p < 0.05; **p < 0.01; ***p < 0.001)

Lasso regression analysis was used to develop the AS-gene-associated prognostic signature, and a prognostic signature model based on seven AS genes was created (Fig. 4A, B). Using the cutoff value of 1.07 set by the Survminer R program (Fig. 4C), patients were separated into two distinct risk groups based on their risk score, survival status, and gene expression heatmap of key predictive AS genes (Fig. 4D). Low-risk patients showed a significant survival benefit (Fig. 4E) (*p* < 0.001). The expression of PRR11, STMN1, RNFT2, CLSPN, MDK, and RNF157 genes in high-risk tumors was significantly higher than low-risk tumors (*p* < 0.001), GHR gene in high-risk tumors was significantly lower compared to low-risk tumors (*p* < 0.001) (Fig. 4F).

### Prognostic value of the riskScore gene signature

**Fig. 5:**
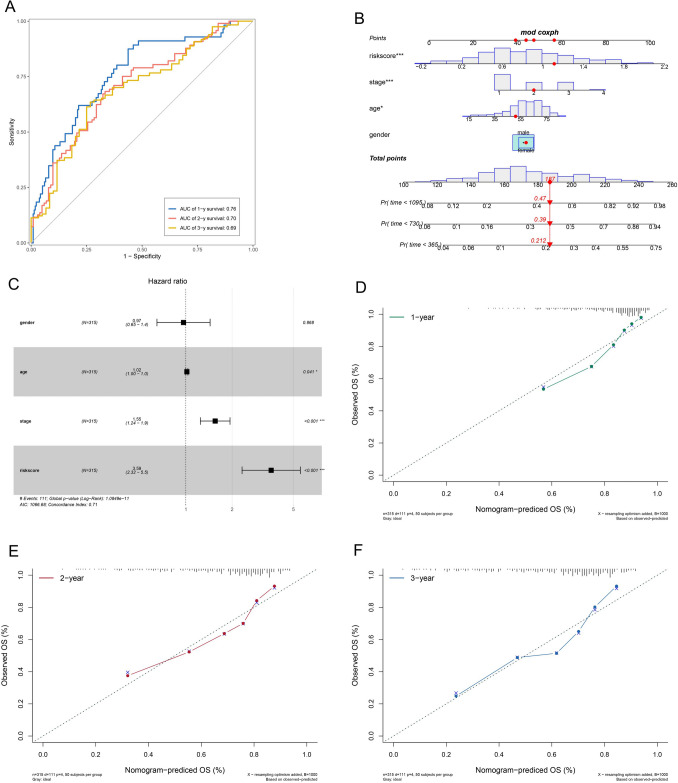
Value for prognosis of the riskScore gene signature. **A** In the TCGA dataset, risk scores were analyzed using time-independent receiver operating characteristic (ROC) analysis to predict OS. **B** The nomogram for HCC patients at 1, 2, and 3 years based on the signature. **C** Forest plot demonstrating the use of multivariate analysis to establish the riskScore as an independent predictive biomarker. **D**–**F** Nomogram calibration curves at 1, 3, and 5 years for the signature

We looked at the risk score model's prognosis accuracy as a continuous variable (Fig. 5A). The AUC of the prognostic model for OS was 0.76 at 1 years, 0.70 at 2 years, and 0.69 at 3 years, indicating that the signature can be used as an accurate predictive tool. The results of a multivariate Cox regression model analysis, which took into account the patients' age, gender, and clinical stage, showed that the riskScore signature may be used as a predictive biomarker for assessing the outcomes of HCC patients on its own (Fig. 5B). We created the nomogram, which combined the riskScore and independent clinical prognostic indicators, to create a clinically relevant mathematical tool for estimating the likelihood that a patient will die. In comparison to the ideal model, the calibration plots showed that the derived nomogram performed well (Fig. 5C–F).

### Validation of the AS gene prognostic signature by ICGC database

**Fig. 6:**
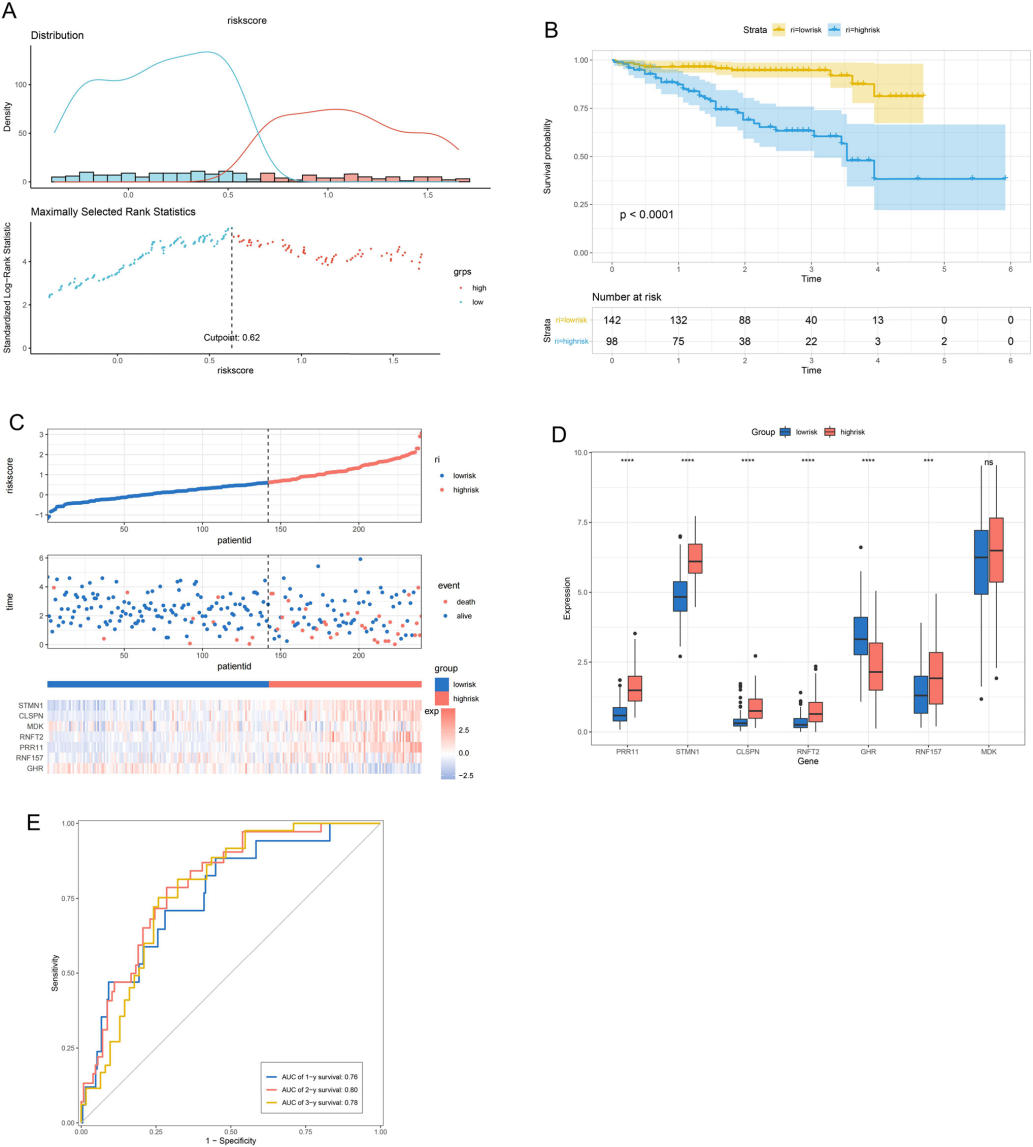
Validation of the AS gene prognostic signature by ICGC database. **A** The optimal cutoff point to dichotomize riskScore into low and high groups. **B** Survival curves for the low-risk and high-risk groups. **C** The risk score, survival status of HCC patients, heatmap of the seven AS genes expression are shown. **D** The seven AS genes expressed in the low- and high-risk groups (*p < 0.05; **p < 0.01; ***p < 0.001). **E** ROC analysis of risk scores for prediction the OS in the ICGC dataset

The AS gene signature was validated using the ICGC database, which contains 240 HCC samples. We split up the patient population into high-risk (*n* = 98) and low-risk (*n* = 142) groups based on the cutoff value of 0.62 (Fig. 6A). The Kaplan–Meier curve revealed that high-risk patients had notably worse OS than other patients (*p* < 0.001) in accordance with the findings from the TCGA dataset (Fig. 6B). Figure 6C displays the risk score, survival status, and heatmap of gene expression for these prognostic AS genes. As shown in Fig. 6D, the expression of the genes PRR11, STMN1, CLSPN, RNFT2, and RNF157 was considerably higher in tumors with high risk compared to others (*p* < 0.001), and the expression of the gene GHR was significantly lower in tumors with high risk compared to tumors with low risk (*p* < 0.001), and MDK expression between high-risk and low-risk tumors showed no difference (*p* > 0.05). Areas under the curve for the signature's value that predicted 1-, 2-, and 3-year OS rates, respectively, were 0.76, 0.80, and 0.78 (Fig. 6E), showing that this prognostic model had acceptable sensitivity and specificity. In general, the performance of the prognostic signature as a classifier was proved to be universally applicable.

### Validation of expression of the AS genes and clinical value of prognostic signature

**Fig. 7:**
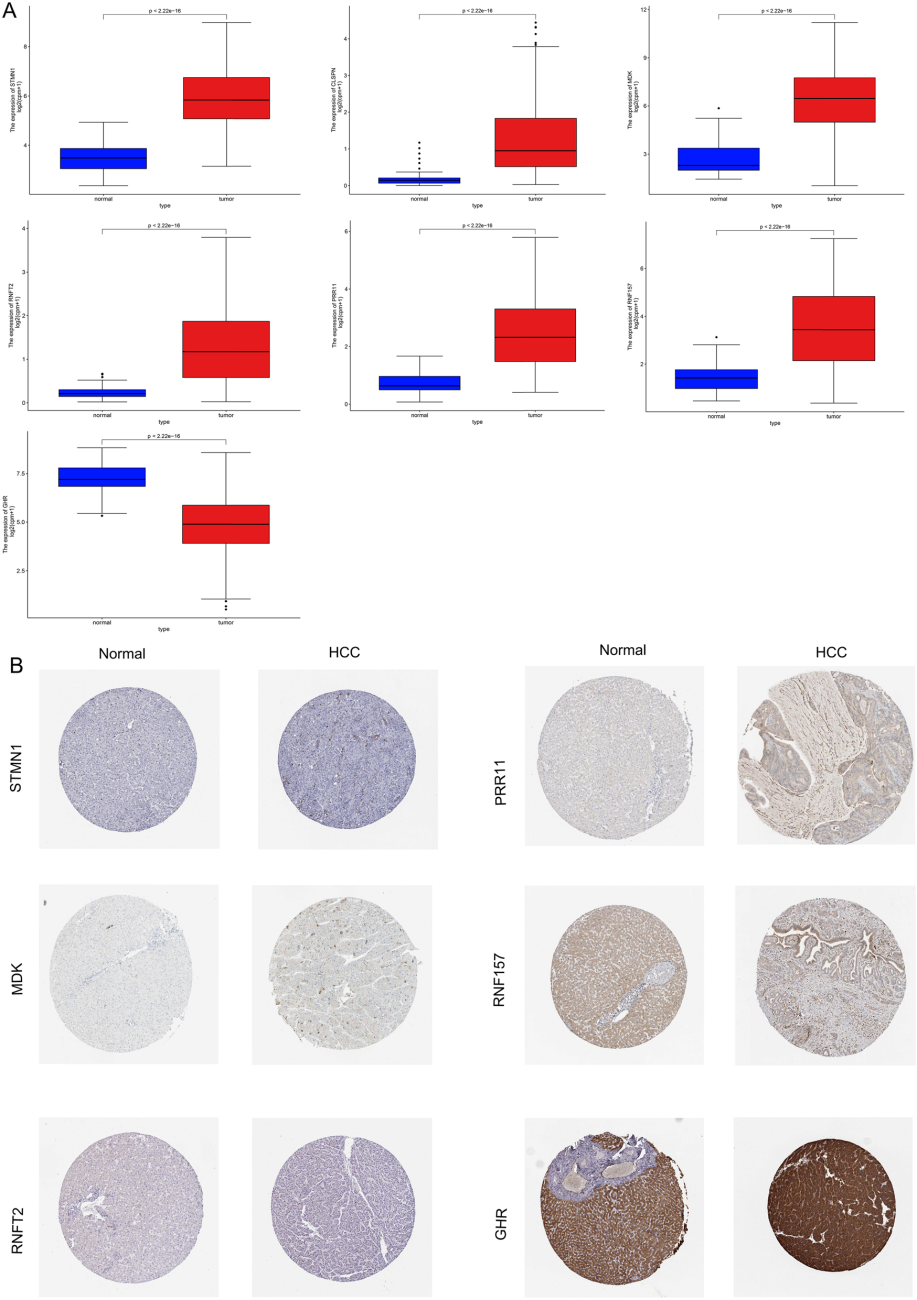
Verification of the AS genes' expression. **A** Comparing the expression levels of seven AS genes in HCC and the equivalent normal tissues using a box plot (TCGA database). **B** The Human Protein Atlas database's profiles of AS genes' protein expression

The expression levels of the seven AS genes were validated in TCGA database (*T* = 315, *N* = 50. The mRNA expression levels of STMN1, CLSPN, MDK, RNFT2, PRR11, and RNF157 were significantly increased in HCC tumor tissue; on the contrary, GHR was significantly decreased compared with normal tissues (Fig. 7A). Protein expression levels were investigated using the Human Protein Atlas database. Figure 7B displays the typical IHC of six genes in tumor and normal pancreatic tissues, with the exception of CLSPN, which is not present in the database (Pictures can be found at v18.proteinatlas.org).

**Fig. 8:**
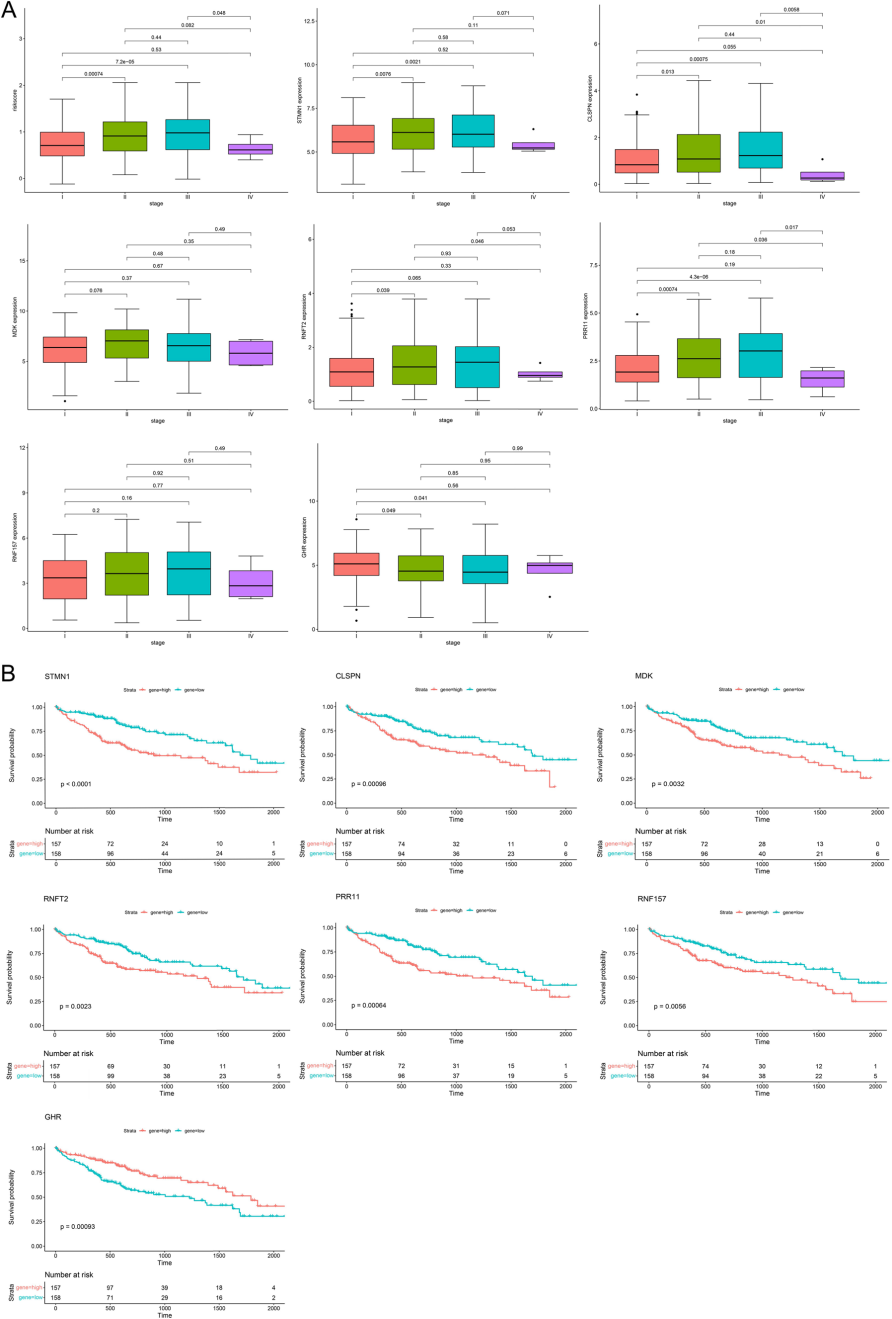
Survival and clinical case characteristic analysis of prognostic signature. **A** Correlations between prognostic signature and clinical features. **B** Kaplan–Meier survival curves for patients with HCC malignancies stratified by seven AS gene expression levels

The relationship between the clinical characteristics of AS genes and Kaplan–Meier survival curve OS analysis was looked into to further assess the clinical value of AS genes. The results showed that high-risk scores and AS genes were positively associated with histological stage in patients with HCC (Fig. 8A). Kaplan–Meier survival curve OS analysis was performed on 315 HCC patient samples, the results showed that high expression of STMN1 (*p* < 0.0001), CLSPN (*p* = 0.00096), MDK (*p* = 0.0032), RNFT2 (*p* = 0.0023), PRR11 (*p* = 0.00064), and RNF157 (*p* = 0.0056) was associated with worse prognosis, low expression of GHR (*p* = 0.00093) was associated with worse prognosis (Fig. 8B).

### The role of prognostic signature in the TME cell infiltration and immunotherapeutic responses

**Fig. 9:**
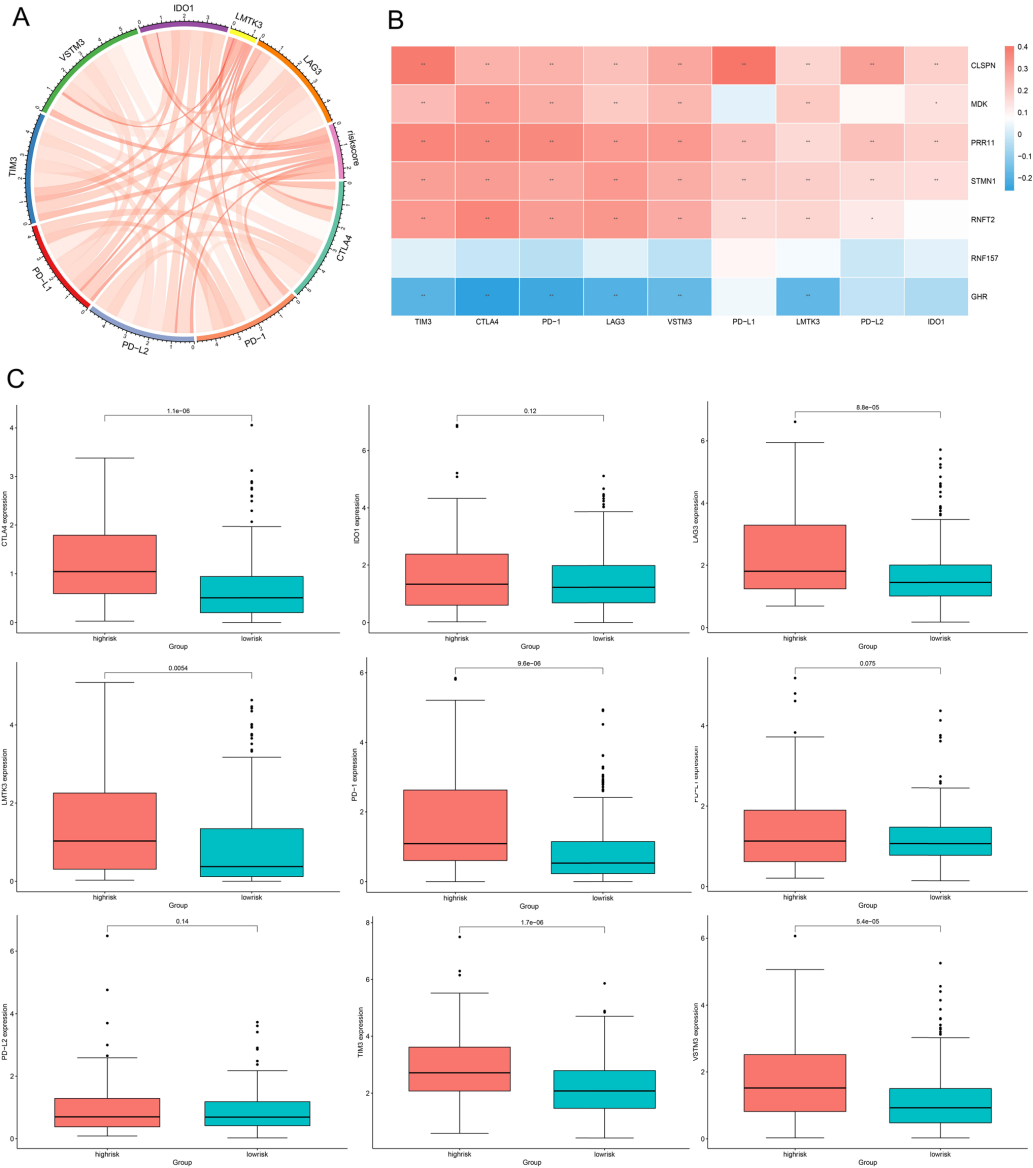
The prognostic signature correlation analysis with immune checkpoint expression. **A** Risk score and immune checkpoint expression correlation chord plots. **B** The correlation between immune checkpoint molecules and the seven AS genes. (*p < 0.05; **p < 0.01). **C** Immune checkpoint expression in low- and high-risk score of HCC

As a new tool in the fight against cancer, immune checkpoint treatment controls the activation of T cells to enhance antitumor immune responses. Therefore, we performed a correlation analysis with the expression of nine immune checkpoint molecules and risk score, discovering a strong positive correlation between the two (Fig. 9A). We discovered that CLSPN, MDK, PRR11, STMN1, and RNFT2 expressions were positively correlated with immune checkpoint molecules, while GHR expression was negatively correlated with immune checkpoint molecules (Fig. 9B). Also, we found that HCC patients with greater risk levels expressed immune checkpoint molecules (CTLA4, LAG3, LMTK3, PD-1, TM3, VSTM3) significantly more frequently than low-risk patients, as shown in Fig. 9C.

**Fig. 10:**
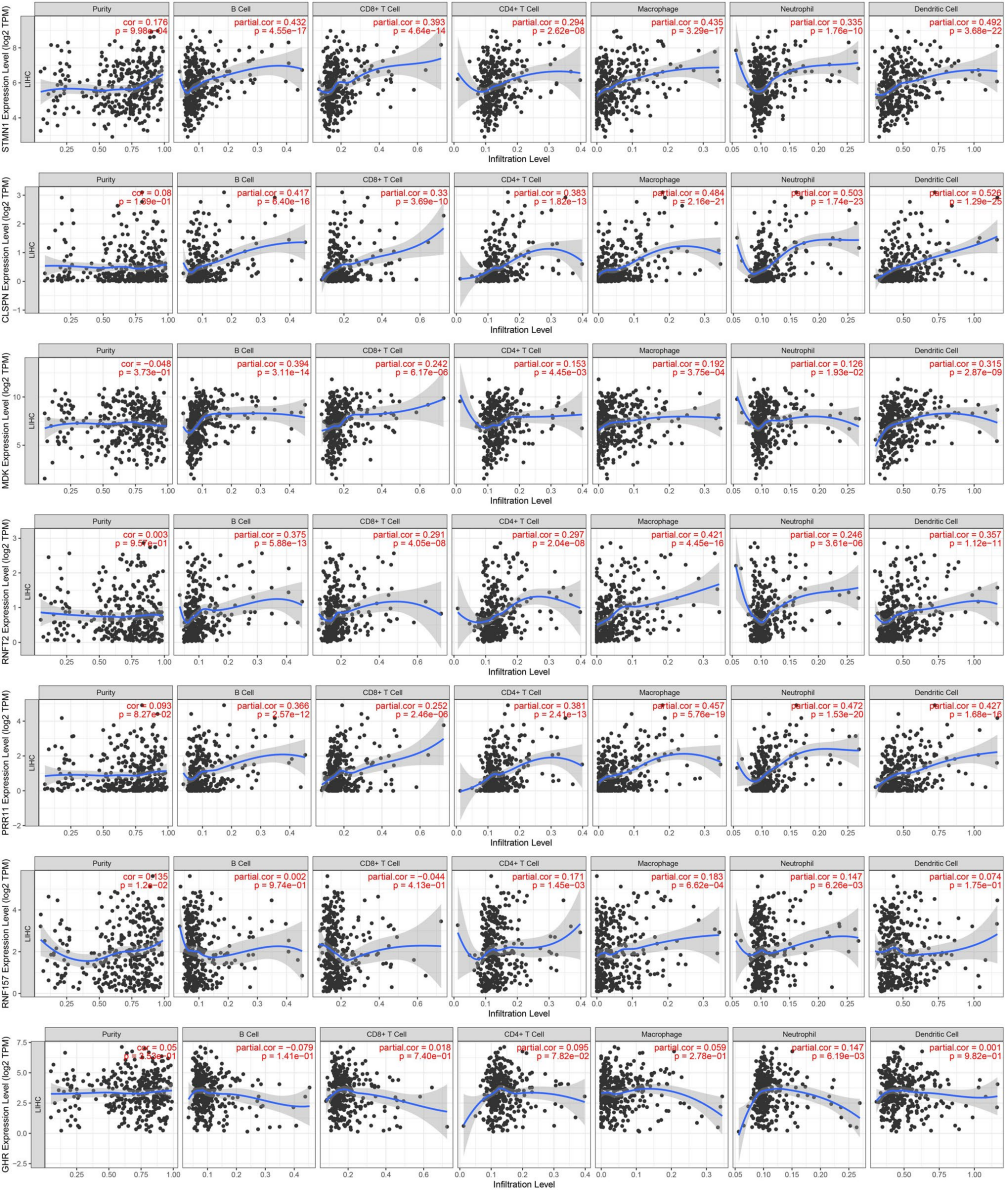
The association between seven AS gene expression and immune cell infiltrations in HCC (TIMER database showed)

The association between the prevalence of immune cells and the seven AS genes was examined using the TIMER database to completely comprehend the properties of immune cells and their relationships with AS genes. STMN1, CLSPN, MDK, RNFT2, and PRR11 were positively correlated with CD4^+^ T cells, CD8^+^ T cells, B cells, neutrophils, macrophages, and dendritic cells (Fig. 10A).

### Survival-associated AS events in HCC

**Fig. 11:**
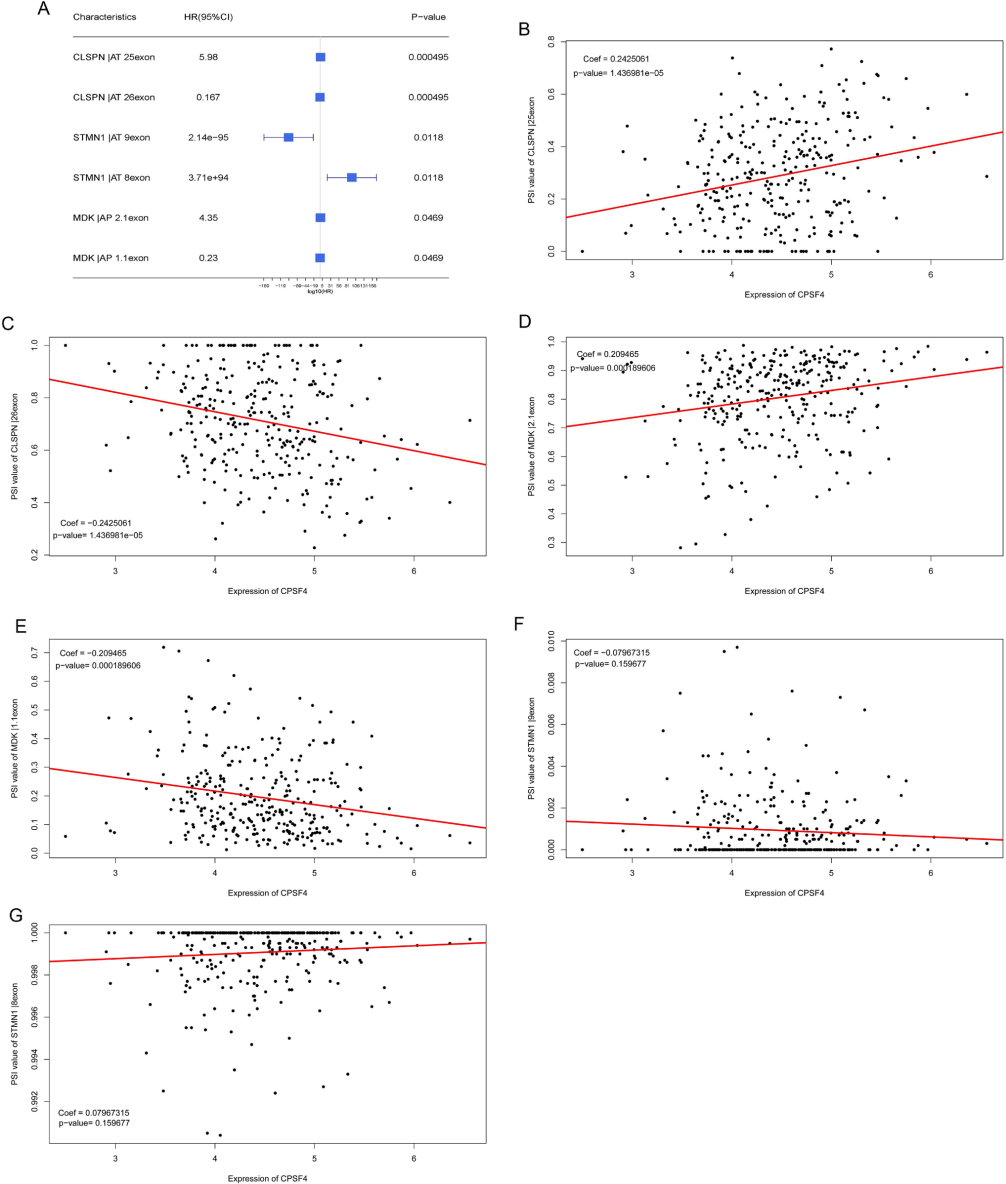
Survival-associated AS events in HCC. **A** Univariate Cox analysis of AS events for seven AS genes. **B**–**G** Correlation between expression of CPSF4 and PSI value of survival-associated AS events

We conducted a univariate Cox analysis of AS events for seven AS genes to identify which AS events are connected to survival. We discovered that the AS events for the CLSPN, STMN1, and MDK genes were related to survival (Fig. 11A). The expression of the CPSF4 gene correlated positively with the AT of CLSPN in the 25 exon and the AP of MDK in the 2.1 exon PSI values of AS events (AT of CLSPN, AT of STMN1, and AP of MDK), as shown in dot plots. But CPSF4 expression was negatively associated with AT of CLSPN in 26 exon and AP of MDK in 1.1 exon PSI value, also CPSF4 expression was not associated with AT of STMN1 in 8–9 exon PSI value (Fig. 11B–G).

## Discussion

Nowadays, the main treatments for HCC are surgical resection and liver transplantation, but due to the frequent recurrence of HCC and the limitations of treatment methods and the poor overall prognosis of HCC patients (Sung et al. 2021), it is challenging to create a reliable predictive model for patients with HCC's overall survival. When compared to models based on single components, prognostic models based on a combination of novel prognostic biomarkers can enhance prognosis (Nault and Villanueva 2015; Torrecilla et al. 2017). In addition, the existing clinical treatment is mainly based on surgical resection, although the prognosis of patients is still poor. Thus, finding a novel clinical feature that is closely associated to the occurrence is urgent, and development of hepatocellular carcinoma to better predict recurrence, metastasis, and prognosis of patients. Recently, more and more studies have concentrated on the analysis of tumor AS genes as high-throughput sequencing technology and computer technology for biological information have advanced (Li et al. 2019; Marzese et al. 2018; Sciarrillo et al. 2020). Studying the mechanism and prognostic value of the AS gene in HCC is crucial since the importance of the AS gene in HCC is still unclear, particularly in HCC prognosis immunotherapy. Previous research has shown that CPSF4 is overexpressed in a number of cancer types and is related to prognosis (Zhang et al. 2021; Wu et al. 2019; Yi et al. 2016). However, previous studies on CPSF4 in liver cancer were limited to its polyadenylation mechanism (Wang et al. 2021b). It is necessary to further explore the regulatory role of CPSF4 on AS gene and its role in malignant phenotype, prognosis, and tumor microenvironment of liver cancer.

Seven AS genes identified by examining TCGA-LIHC data (STMN1, CLSPN, MDK, RNFT2, PRR11, RNF157, and GHR) were employed in this investigation to predict the prognosis of HCC with greater accuracy. STMN1 is a cytosolic phosphoprotein that regulates microtubule dynamics in response to cellular needs, and plays an important role in mitotic spindle formation and cell division (Rubin and Atweh 2004; Hu et al. 2020). The methylation of STMN1 is associated with the prognosis of HCC, and the expression of STMN1 is closely related to the change of m6A (Zhang et al. 2022). CLSPN, as the gene encoding Claspin protein, plays an important role in key cellular events such as checkpoint activation after DNA damage, DNA replication and replication stress response, DNA repair and apoptosis (Azenha et al. 2017, 2019). MDK is a growth factor, which participates in various physiological processes of organisms. Its overexpression in tumor tissues promotes the growth, migration, induction of EMT and multidrug resistance of tumor cells (Filippou et al. 2020; Hu et al. 2021). RNFT2 as an inhibitor of inflammation targeting IL-3 cytokine receptor IL-3Rα degradation, it may play an important role in the innate immune response chain of lung cancer (Tong et al. 2020). GHR is a membrane-bound receptor belonging to the class I cytokine receptor superfamily, which has been implicated in the development of many types of cancer (Zhu et al. 2022).

An independent predictive factor for HCC patients was found to be a seven-gene-based risk score after univariate and multivariate analysis of the clinical data from the TCGA-LIHC. Patients in the high-risk group had a considerably worse outcome than those in the low-risk group. Our model shows a high AUC between 1, 2, and 3 years and can accurately predict short-term survival in HCC patients, suggesting that our AS gene profile has a significant predictive advantage. In this work, a predictive model was created for predicting overall survival in HCC patients by identifying a novel five-gene signature through thorough data analysis. Yet there are several gaps in our investigation. First, it still has to be supplemented with an external validation dataset. Second, the seven genes' expression and prognostic implications at the protein level were not examined in this study. Third, more clinical validation is necessary to confirm the scoring model's dependability. The results of this investigation must, thus, be confirmed by subsequent clinical studies.

## Conclusion

As a result of our research, seven CPSF4-related AS genes were shown to be predictive of the prognosis for patients with liver cancer. We then looked into the associations between prognostic factors including immune cell infiltration and tumor microenvironment. In addition to offering useful indicators and novel prognostic treatment targets, our findings will contribute to understanding the regulatory role of CPSF4 in AS events in liver cancer and the function of AS genes in malignancies.

## Supplementary Information

Below is the link to the electronic supplementary material.

Supplementary file1 (CSV 377 KB)

## Data Availability

Online repositories contain the datasets used in this investigation. The article/Supplementary Material contains the name(s) of the repository(s) and the accession number(s).
